# Non-small cell lung carcinoma in an adolescent manifested by acute paraplegia due to spinal metastases: a case report

**DOI:** 10.1186/1752-1947-5-486

**Published:** 2011-09-28

**Authors:** Ulrike Ackert, Dieter Haffner, Carl Friedrich Classen

**Affiliations:** 1 University Children's Hospital Rostock, Ernst-Heydemann-Strasse 8, D-18057 Rostock, Germany; 2 Department of Pediatric Kidney, Liver, and Metabolic Diseases, Hannover Medical School, Carl-Neuberg-Strasse 1, D-30625 Hannover, Germany

## Abstract

**Introduction:**

Bronchial carcinomas in childhood and adolescence are extremely rare; only individual cases have been reported previously.

**Case presentation:**

We report on a 16-year-old Caucasian German boy with non-small cell lung carcinoma (squamous cell non-small cell lung carcinoma) stage IV, T4N2M1, without epidermal growth factor receptor overexpression and/or mutation or k-ras mutation. He presented with paraplegia due to spinal metastases of the bronchial carcinoma. No familial predisposition or toxin exposure was identified. Treatment following adult protocols consisted of surgical intervention for spinal metastases, first-line cisplatinum and gemcitabine, irradiation and second-line docetaxel. After a transient response our patient experienced disease progression and died about 10 months later.

**Conclusion:**

Response and survival in our 16-year-old patient were similar to adult patients with stage IV non-small cell lung carcinoma.

## Introduction

Lung cancer is the leading cause of cancer-related mortality in adults in many countries, especially in smokers [1,2]. There are small cell and non-small cell lung cancers (NSCLC). NSCLC are subdivided into adenocarcinomas (about 50%), squamous cell (about 20%), large cell (about 10%), and otherwise not defined carcinomas [1]. The TNM staging system is closely associated with prognosis. Metastatic disease, defined as stage IV, accounting for 10% to 20% of cases, has a median survival time of nine months and a five-year survival of about 1% [2]. The peak age for NSCLC is 50 years to 60 years [2]. Recently, several molecular characteristics of NSCLC, including p53 mutations, epidermal growth factor receptor (EGFR) mutations with subsequent dysregulation of the ras/raf kinase pathway and k-ras mutations, have been defined [3], leading to some targeted therapy approaches. Their benefit is still not quite clear [4,5].

The therapy options--surgery, chemotherapy, irradiation and targeted therapy--are usually applied according to the TMN staging and the molecular signature [3-5]. Tumors should be resected primarily whenever possible [6]. Unresectable tumors imply a palliative condition. Combination first-line chemotherapy is used to obtain maximum efficacy, eventually combined with radiotherapy. In second-line treatment, monotherapy is proposed for patients in an appropriately good condition [4,6].

For first-line therapy, cisplatinum-based chemotherapy plus radiotherapy is superior to radiotherapy or chemotherapy alone [6,7]. Several agents have been applied in addition to cisplatinum, for example paclitaxel, docetaxel, gemcitabine, pemetrexed, vinorelbine and irinotecan. Gemcitabine is regarded as first choice in squamous cell carcinomas by most authors [7]. For second-line therapy, monotherapy, for example with docetaxel, is common. Usually, an aggressive therapy would now be inappropriate [7].

In the last years, targeted therapies have shown antitumoral effects. Most targets are related to EGFR or the raf/ras kinase signaling, since deregulation of this pathway is common in NSCLC [3,7]. Both EGFR-targeted antibodies, for example cetuximab, and kinase inhibitors, like erlotinib, have a low toxicity, however, their efficacy depends on the signaling pathway deregulation [3,7]. Antibodies against the vascular-endothelial growth factor receptor (VEGFR) are efficient in non-squamous cell tumors [1].

Lung carcinomas in patients below 20 years of age are extremely rare. Here, we report the case of a 16-year-old boy with a non-small cell, squamous cell bronchial carcinoma T4N2M1 initially presenting with paraplegia due to spinal metastases. In spite of all therapeutic measures performed according to adult treatment protocols our patient experienced only a transient response, followed by disease progression, and died about nine and half months after diagnosis.

## Case presentation

Our patient was the second child of non-consanguineous healthy parents, both Caucasian (ethnically German); his sister was healthy. Three cases of carcinomas, including a bronchial carcinoma, had been observed in grandparents and an aunt.

He was born after an uneventful pregnancy and showed normal development except for mild attention deficit disorder. He never smoked nor abused other substances, nor was he exposed to tobacco smoke, chemicals or irradiating toxins.

At the age of 16 years, eight weeks before initial presentation, he experienced an episode of back pain followed four weeks later by unproductive cough with minimal hematoptysis. A chest X-ray showed a discrete central infiltrate. Two weeks before admission he had several episodes of back pain. Finally, the boy had experienced a numb feeling in the lower body half from about thoracic vertebra (Th) seven downwards, loss of strength and insecure gait for two days prior to admission. Pain sensitivity and tendon reflexes were preserved. Strength was grade four in his legs and feet. A sharpened breath murmur of amphoric type was heard. Otherwise, the boy was in a stable condition with a normal examination status.

**Figure 1:**
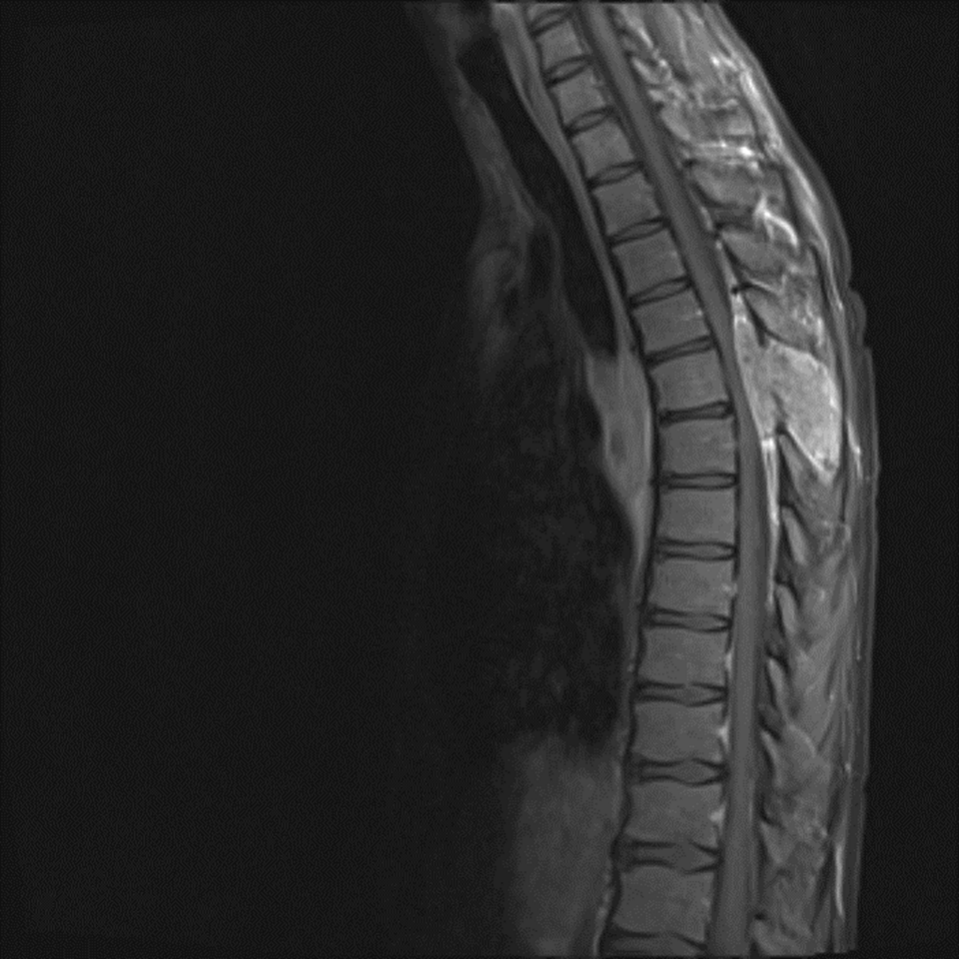
**MRI of the thoracic spine, in sagittal view, T1-weighed image with contrast medium enhancement**. The tumor leads to significant myelocompression, and grows infiltratingly into the surrounding tissue.

**Figure 2:**
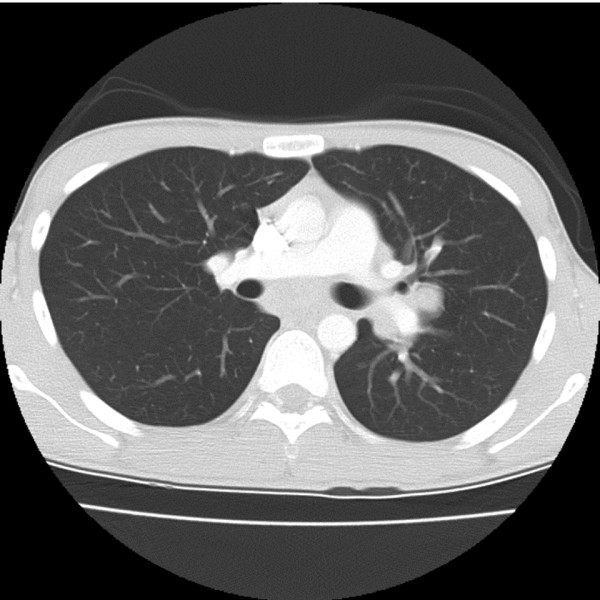
**CT scan of his lungs, showing a large central space occupying lesion surrounding both main bronchi**.

Magnetic resonance imaging (MRI) of his spine, followed by computed tomography (CT) of his chest (Figures 1 and 2) showed an acute transverse myelocompression at Th5 by a tumor with intraspinal extensions, and a central intrapulmonary lesion infiltrating both main bronchi. An emergency laminectomy with subtotal tumor resection was performed with stabilization by a fixateur interne. A histological analysis showed a non-small cell squamous bronchial carcinoma (NSCLC), EGFR amplification and k-ras mutation negative. Staging included a bronchoscopy with biopsy of an intrabronchial tumor portion, a bone scan, MRI and CT. An inoperable lung tumor, with metastases at Th5 and in his os sacrum and left scapula, was diagnosed, in other words, stage IV, T4N2M1. A familiar p53 mutation and a germ cell tumor were excluded. Complete laboratory and clinical workup showed no infections, immunodeficiency or drug addiction.

Treatment was initiated according to current recommendations for adults. Six cycles of intravenous cisplatinum (70 mg/m^2^/d, day one) and gemcitabine (1000 mg/m^2^/d, day one and day eight) were given three-weekly, with a bisphosphonate (pamidronate, 30 mg) every four weeks. New generation EGFR or signaling inhibitors were not applied since k-ras mutation and EGFR overexpression and/or mutation were negative.

After four weeks, local progression of the sacral metastasis led to ischial nerve irritation requiring extended analgetic medication (opioids plus pregabalin). Fractionated sacral irradiation with 30 Gy soon induced analgesia. Clinical tumor progress at Th5 developed at about three months, confirmed by MRI, which led to neurosurgical resection of a regrown metastasis. Since it was not feasible to separate irradiation of the main bronchial tumor and the area of Th5, combined fractionated irradiation with 30 Gy was subsequently performed.

Toxicity of the six cycles of cisplatinum and gemcitabine over the first four months following diagnosis consisted of vomiting (WHO III°), infection (WHO II°), tricytopenia (WHO III°) and cachexia (WHO III°), requiring home parenteral nutrition.

Now, a chest CT scan showed shrinkage of the main bronchial tumor but also appearance of pleural lesions, likely to represent metastases. Thus it appeared inappropriate to proceed with gemcitabine and cisplatinum, since our patient wanted a less toxic therapy.

In accordance with recommendations for second-line therapy in adults, the boy received docetaxel (75 mg/m^2^/d every three weeks) as monotherapy for four and a half months after diagnosis. However, disease progression developed one month later with new spinal metastases at Th12, L1, L2 and L4. Another course of palliative irradiation was given for analgesia. Besides cachexia, the boy suffered from febrile chills and cough and a pleural effusion. About seven months after diagnosis, chemotherapy was discontinued to preserve quality of life as much as possible.

About eight months after diagnosis, the boy presented with double vision, headache and diabetes insipidus. A CT scan of his head revealed numerous brain metastases. Palliative dexamethasone was initiated.

Further multimodal palliative care was provided and our patient lived until about nine and a half months after diagnosis and died at home.

## Discussion

Since 1876, when McAldowie first described a case of lung cancer in a five-month-old child [8], the disease has only occasionally been reported in children and adolescents. Published series include different histological entities including carcinoids, mucoepidermoid carcinomas, inflammatory pseudotumors, and others; only a minority are squamous cell carcinomas [9-11]. In 1977 and 2003, two series of 24 and 92 patients, respectively, with lung cancer below 40 years were published, with only one patient below 20 years in each. Their prognosis was similar to that of older patients [12,13]. Analyzing 26 patients below 30 years, Mizushima *et al. *[14] found a more favorable prognosis in the young patients, however, there was a low incidence of squamous cell carcinoma and a predominance of stage I.

The largest series of pediatric lung cancer was collected in a 1983 meta-analysis [15]. In 151 malignant lung tumors, beside many carcinoids and lymphomas, 47 were bronchogenic carcinomas, including six squamous cell tumors.

In conclusion, the published literature on pediatric squamous NSCLC is too sparse to draw any clear conclusion as compared with the older population.

In our case, several questions remain unanswered. First, why did the boy develop the disease in the first place? Neither the analysis of the tumor itself, nor the anamnestic or clinical workup gave any indication as to cause; in particular, no toxin exposure, immunodeficiency or familial disposition could be identified.

The second question is whether the exceptionally young age of our patient was associated with any special disease features. We found that histology, genetics, distribution, type of response and time course of the disease were similar to what is observed in older patients. Finally, it has to be asked whether any other treatment options might have been effective in our patient. More than six cycles of cisplatinum and gemcitabine chemotherapy in combination with irradiation, as first-line standard therapy for NSCLC patients, are rarely tolerated. Equally, the irradiation given as palliative focal treatment of painful bone metastases followed by tumor field irradiation after tumor shrinkage by initial chemotherapy represent a common approach in adults. So it may only be speculated whether further addition of a targeted drug might have been beneficial. Randomized studies indicate that drugs targeting the EGFR or ras/raf kinase pathway are only effective in cases with deregulation of these pathways [3]. Other treatment modalities or strategies that might have helped the boy do not, to our best knowledge, exist.

## Conclusion

Taken together, in the case reported here, we observed a stage IV NSCLC in a 16-year-old boy, in which the course of the disease was as may be expected in typical cases contracting the disease at an older age.

## Consent

Written informed consent was obtained from the mother (legal guardian) of our patient for publication of this case report and any accompanying images. A copy of the written consent is available for review by the Editor-in-Chief of this journal.

## Competing interests

The authors declare that they have no competing interests.

## Authors' contributions

UA analyzed and interpreted the patient data regarding the clinical presentation. DH added particular background information and was a major contributor in writing the manuscript. CFC performed basic organization of the writing and clinical data collection. All authors read and approved the final manuscript.
